# Hesperetin Alleviated Experimental Colitis via Regulating Ferroptosis and Gut Microbiota

**DOI:** 10.3390/nu16142343

**Published:** 2024-07-19

**Authors:** Jinzhi Wang, Yuanyuan Yao, Ting Yao, Qingmiao Shi, Yifan Zeng, Lanjuan Li

**Affiliations:** 1 State Key Laboratory for Diagnosis and Treatment of Infectious Diseases, National Clinical Research Center for Infectious Diseases, National Medical Center for Infectious Diseases, Collaborative Innovation Center for Diagnosis and Treatment of Infectious Diseases, The First Affiliated Hospital, Zhejiang University School of Medicine, 79 Qingchun Rd., Hangzhou 310003, China; 2 Jinan Microecological Biomedicine Shandong Laboratory, Jinan 250000, China

**Keywords:** ulcerative colitis, ferroptosis, hesperetin

## Abstract

Hesperetin (HT) is a type of citrus flavonoid with various pharmacological activities, including anti-tumor, anti-inflammation, antioxidant, and neuroprotective properties. However, the role and mechanism of HT in ulcerative colitis (UC) have been rarely studied. Our study aimed to uncover the beneficial effects of HT and its detailed mechanism in UC. Experimental colitis was induced by 2.5% dextran sodium sulfate (DSS) for seven days. HT ameliorated DSS-induced colitis in mice, showing marked improvement in weight loss, colon length, colonic pathological severity, and the levels of TNFα and IL6 in serum. A combination of informatics, network pharmacology, and molecular docking identified eight key targets and multi-pathways influenced by HT in UC. As a highlight, the experimental validation demonstrated that PTGS2, a marker of ferroptosis, along with other indicators of ferroptosis (such as ACSL4, Gpx4, and lipid peroxidation), were regulated by HT in vivo and in vitro. Additionally, the supplement of HT increased the diversity of gut microbiota, decreased the relative abundance of Proteobacteria and Gammaproteobacteria, and restored beneficial bacteria (Lachnospiraceae_NK4A136_group and Prevotellaceae_UCG-001). In conclusion, HT is an effective nutritional supplement against experimental colitis by suppressing ferroptosis and modulating gut microbiota.

## 1. Introduction

Ulcerative colitis (UC) is a type of inflammatory bowel disease (IBD) that has prevailed with the westernization of society around the world, for instance, the improvements in diagnosis technology [1] and increasing adoption of a sedentary lifestyle [2] in recent decades. The prevalence of IBD is 0.12–0.25‰ in western regions and is estimated to progress up to 1% by 2030 [1], which brings about a heavy economic burden on the global healthcare system [3]. UC typically manifests with persistent or repetitive abdominal pain and diarrhea with pus–blood, accompanied by systemic symptoms including rectal tenesmus and fever, which can disrupt a normal life [4,5]. Despite the unclear mechanism of UC, recent studies suggested that gut dysbiosis, ferroptosis, and dietary patterns play an important role in the pathogenesis of UC [6,7,8]. The current therapies of UC mainly consist of conventional medicine (5-aminosalicylic acid, corticosteroids, and immunosuppressants) and emerging biological agents such as anti-tumor necrosis factor alpha (TNFα) inhibitors [9,10]. It is recommended that those who respond poorly to conventional medicine treatment receive biological therapies [11]. However, only approximately 50% of patients with UC could achieve remission after 12 months of administration of biological therapies [10]. In addition, long-term application of these medicinal treatments could lead to many adverse effects [9,12]. Therefore, it is important to seek efficient and safe therapies for UC.

Hesperetin (HT), a natural flavonoid derivative, is widely found in many citrus fruit species, including lemons, grapefruit, and oranges [13]. HT is derived from the hydrolysis of hesperidin [14]. Existing non-clinical studies have demonstrated that HT exerts various bioactive effects, including anti-tumor [15], anti-inflammation [16], antioxidant [17], and neuroprotective properties [18]. Patients with anitis and/or proctitis received treatment with bergamot-derived gel and mesalazine and showed a satisfactory clinical response. However, the extent to which the bergamot-derived flavonoids contributed to the improvement of the disease remains unclear due to the lack of a suitable reference group [19]. Several studies indicated that HT ameliorated experimental colitis in rodents [20,21], suggesting that HT is a potential therapeutic strategy for UC; nevertheless, the mechanism behind how HT protects against UC remains unclear. Additionally, the extract of orange juice, which is HT-rich, decreased ROS, lipid peroxidation, and iron-induced oxidative damage in vitro [22,23], implying that HT might intervene in ferroptosis. Ferroptosis is an iron-dependent form of programmed cell death, characterized by shrunken mitochondria, lethal ROS, and lipid peroxidation, which is markedly different from other forms of cell death, such as apoptosis, necrosis, and pyroptosis [24]. Recent studies demonstrated that iron deposition, excessive ROS and lipid peroxidation, and alterations in ferroptosis-related genes were observed in patients with UC [25,26]. Meanwhile, the inhibition of ferroptosis remarkably alleviated symptoms and pathohistological severity of colitis in rodents [27], suggesting that ferroptosis could be a potential target of UC.

Recently, several researchers have revealed that obvious gut dysbiosis exists in patients with UC [28,29] and experimental colitis [30], which participates in intestinal immune disorders and the progression of the disease. The restoration of intestinal homeostasis is beneficial for the treatment of UC [31]. Although it was found that the HT-rich extract of citrus unshiu peel modulated the gut microbiome and improved the intestinal barrier [32], whether HT regulates the gut microbiota in experimental colitis remains elusive. Here, our work aimed to unveil the effect of HT on DSS-induced colitis by a combination of informatics, network pharmacology, molecular docking, microbiome, and experiments in vivo and in vitro.

## 2. Methods

### 2.1. Reagents and Instruments

HT was purchased from MedChemExpress (Shanghai, China). Dextran sulfate sodium salt (DSS, M.W 40,000) and carboxymethyl cellulose sodium (CMC-Na) were bought from Macklin (Shanghai, China). Anti-GAPDH Mouse Monoclonal antibody (mAb) was bought from Servicebio (Wuhan, China). ACSL4 rabbit mAb was from ABclonal (Wuhan, China). GPX4 mouse mAb and PTGS2 mouse mAb were both from Proteintech (Wuhan, China). HRP-labeled Goat Anti-Rabbit IgG (H + L) and HRP-labeled Goat Anti-Mouse IgG (H + L) were from Beyotime (Shanghai, China).

### 2.2. Animal Experiments

All animal experiments in our study were approved by The Tab of Animal Experimental Ethical Inspection of the First Affiliated Hospital, Zhejiang University School of Medicine (Reference Number: 2023-1354, approval date: 28 August 2023). Moreover, the animal experiments were in line with laboratory animal use and care principles proposed by the European Community guidelines.

Twenty-four C57BL/6 mice (male, aged 6 weeks, weighing 20 ± 2 g, specific pathogen-free) were bought from Hangzhou Ziyuan Experimental Animal Technology Co., Ltd., Hangzhou, China. The mice were randomly assigned to the control group, Dextran Sulfate Sodium Salt (DSS) group, and DSS + HT group and there were equally eight mice in each group. The mice were kept in a constant-temperature environment (22 °C) with an alternation of 12 h light/dark cycle. All mice were adjusted to the new environment for at least two weeks before the start of the study.

Once the study started, the mice in the DSS group and DSS + HT group received drinking water with 2.5% DSS and the mice in the control group were given free access to drinking water. In addition, the mice in the DSS + HT group were intragastrically administered with HT [100 mg/(kg·day)] in 0.5% CMC-Na by a gavage needle for 7 days, while the mice in the other two groups received an equal volume of CMC-Na by gavage. The weights of mice were measured, and the stool characters were recorded; meanwhile, the disease activity index (DAI) [33] (Table S1) of each mouse was evaluated daily. On the eighth day, all mice were given normal drinking water and then sacrificed. The sacrifice was executed by inhalational anesthesia with isoflurane, followed by dislocation of the cervical vertebra; blood and colon tissues were collected for further analysis.

### 2.3. Histopathological Analysis

Approximately 1 cm of the colon was collected at 1 cm above the anus, then fixed in neutral universal tissue fixative fluid for at least 24 h and paraffin-embedded. The paraffin-embedded tissues were cut into 4 μm-thick sections before hematoxylin and eosin (HE) staining. To evaluate the severity of colitis in the mice, the histopathological score was determined with the widely used criteria [34] shown in Table S2.

### 2.4. Immunohistochemical (IHC) Staining

Colon paraffin sections were dewaxed to water by dimethylbenzene, ethanol, 95% ethanol, 75% ethanol, and water in turn, followed by antigen repair in a citrate antigen retrieval solution (pH 6.0) for 5 min in a microwave with medium heat. Then endogenous peroxidase inactivation was performed via incubation with 3% hydrogen peroxide solution for 25 min at room temperature. The PTGS2 mAb (1:1000 dilution) was used to submerge tissue sections at 4 °C overnight after blocking by 3% bovine serum albumin for 30 min. The PTGS2 mAb (1:1000 dilution) was used to submerge tissue sections at 4 °C overnight. After the incubation with the corresponding secondary antibody, tissue sections were developed in color with 3,3′-diaminobenzidine tetrahydrochloride and the pictures were taken by an Olympus BX53 fluorescent upright microscope (Olympus, Tokyo, Japan).

### 2.5. 16S rRNA Gene Sequencing

Isolation of microbial DNA of colon contents was performed with a Stool Genomic DNA Extraction Kit (Solarbio, Beijing, China). An equal amount of DNA from each sample (n = 5) was used for the amplification of the V3–V4 region of the 16S rRNA gene with the primer: 27F (5′-AGAGTTTGATCCTGGCTCAG-3′) and 338R (5′-TGCTGCCTCCCGTAGGAGT-3′). The high-throughput sequence was performed on IlluminaMiSeq PE 300 (Illumina, San Diego, CA, USA) and 16S rRNA sequencing reads were acquired. The reads were combined by FLASH.2.11, with quality control and filtration simultaneously. The following microbial bioinformatic analysis was conducted on the online platform of Majorbio Cloud Platform (www.majorbio.com, accessed since 10 December 2023) [35].

### 2.6. ELISA

To acquire serum, the blood of mice was collected using the capillary tube from the ophthalmic venous plexus and placed at room temperature for one hour before centrifugation at 3000 rpm, 4 °C for 15 min. For the colitis model in vitro, the supernatant of cells in different groups was collected for detection. The inflammatory factors (TNFa and IL6) were examined following the instructions of the ELISA Kits (Liankebio, Hangzhou, China).

### 2.7. Network Pharmacology

#### 2.7.1. Screening UC-HT Target Genes (UCHTTGs)

Firstly, the target genes of HT were acquired from the Encyclopedia of Traditional Chinese Medicine (ETCM) [36], Comparative Toxicogenomics Database (CTD) [37], and SwissTargetPrediction (STP) [38], respectively (Table 1). The term “hesperetin” was applied to search for the target genes of HT in ETCM and CTD. A simplified molecular input line entry system (SMILES) form of HT (Compound CID: 72281) obtained from Pubchem [39] was keyed in STP for the prediction of target genes. Repeated target genes of HT from the three databases were removed and the others were regarded as target genes of HT (HTTGs).

Secondly, to screen the target genes of UC, retrieval was performed in DisGeNET [40], GeneCards [41], and Gene Expression Omnibus (GEO) [42] databases, respectively, with “Ulcerative colitis” as the search term. For target genes of UC from GeneCards, those with relevance scores ≥ 3.0 were chosen. To obtain the UC target genes in the GEO database, two datasets (GSE65114 and GSE87466) were employed, followed by normalization with “limma” R package and differential expression gene (DEG) analysis with the criteria |fold change| ≥ 1.5 and adjusted. *p* < 0.05. Duplicates of DEGs from the two datasets were removed and target genes for UC in GEO were obtained. The genes that existed in more than one database were defined as target genes in UC (UCTGs). Then, the intersection of HTTGs and UCTGs was recognized as the target genes of HT in UC (UCHTTGs), visualized by jvenn [43].

#### 2.7.2. Protein–Protein Intersection (PPI) Network Establishment and Core Targets Identification

The list of UCHTTGs was input for the construction of the PPI network with the reassessment of protein names in the STRING 12.0 database [44]. “*Homo sapiens*” was selected and the minimum required interaction score was set as 0.7, which indicated a high confidence in protein interactions. Then, the PPI network was exported and visualized via Cytoscape 3.9.1 software [45]. The node’s score was calculated by the plug-in cytoHubba [45], and the top ten targets ranked by closeness, degree, Maximal Clique Centrality (MCC), Maximum Neighborhood Component (MNC), and radiality value were obtained. Furthermore, an Upset diagram drawn by Xiantao Academic Tools [46] showed that the intersection of the top-ten targets of these five ranking methods (closeness, degree, MCC, MNC, and radiality) were identified as the hub genes of HT in UC.

#### 2.7.3. The Gene Ontology (GO) and KEGG Enrichment Analysis

The GO and KEGG enrichment analysis of UCHTTGs was performed by “clusterProfiler” and “org.Hs.eg.dbo” R package with pvalueCutoff = 0.05 and the visualization of the results was completed with the usage of “ggplot2” R package in R 4.3.1 software.

#### 2.7.4. Molecular Docking between HT and Its Target Proteins

For molecular docking, firstly, the HT 3D structure in sdf file format was acquired from Pubchem [39] and the core target proteins in pdb or cif file format were downloaded from the Protein Data Bank (PDB) [47]. The target protein in cif file format was transformed to the pdb file format via OpenBabel 3.1.1 software [48]. Molecular docking between HT and its target proteins was performed via CB-DOCK2 [49]. Specifically, the pdb files of HT and its target protein were uploaded to the CB-DOCK2 website, followed by searching cavities and docking within the selected CurPockets. The molecular docking results included binding cavity volume, center of cavities, docking size, 3D view of docking, and the Vina Score (kcal/mol).

### 2.8. Real-Time Quantitative Polymerase Chain Reaction (RT-qPCR)

Isolation of the total RNA of the distal colon tissues or cells was conducted with the application of AFTSpin Tissue/Cell Fast RNA Extraction Kit for Animal (ABclonal, Wuhan, China) and an equal mass of RNA of each sample was reverse-transcribed to complementary DNA with PrimeScript™ RT reagent Kit (Takara, Beijing, China). Then, RT-qPCR analysis was performed with an ABI ViiA 7 real-time fluorescence quantitative PCR instrument (Life Technologies, New York, NY, USA). The names and sequences of corresponding primers are listed in Table 2. The relative expression of target genes was calculated using the 2^−ΔΔCT^ method, with glyceraldehyde-3-phosphate dehydrogenase (GAPDH) as an endogenous control gene.

### 2.9. Western Blot Analysis

Extraction of colonic tissue or RAW264.7 cell protein was performed by the buffer (RIPA: Protease inhibitor cocktail for general use, 100× for 100:1) and 1/4 volume of SDS-PAGE Sample Loading Buffer (5×) to the buffer was added before heat at 100 °C for 5 min. The sodium dodecylsulfate-polyacrylamide gel electrophoresis (SDS-PAGE) Gel Quick Preparation Kit (Beyotime, Shanghai, China) was used for appropriate SDS-PAGE gels and the protein of the samples was separated and then transferred onto the Polyvinylidene Fluoride (PVDF) membrane. The membrane was immediately washed with Tris Buffered Saline containing 0.1% Tween-20 (TBST) for 2–3 min and blocked with the quick blocking buffer for Western blot for 15 min. Afterward, the membrane was incubated with the primary antibody at 4 °C overnight. The following day, the membrane was washed with TBST three times for 10 min each time before incubation with the corresponding secondary antibody. The expression of proteins was visualized by a super-sensitive ECL chemiluminescent substrate on the hemiScope3300PRO (ChemiScope, Shanghai, China).

### 2.10. Transmission Electron Microscope

To detect the morphology of mitochondria, fresh colon tissues were placed into electron microscopy fixative with 2.5% glutaraldehyde (pH 7.0–7.5, Wuhan, China) and quickly cut into a 1 mm × 1 mm × 1 mm piece. After fixation for 24 h, the fixative was absorbed and the sample was washed by PBS three times, for 15 min each time. Then the sample was post-fixed, dehydrated, penetrated, embedded, sliced, and stained. The images of samples were taken with HT7650 (HITACHI, Tokyo, Japan).

### 2.11. Malondialdehyde (MDA) and Superoxide Dismutase (SOD) Examination

The detection of MDA and SOD in colon tissues was conducted according to the instructions (Biosharp, Hefei, China), and the BCA Protein Assay Kit (Beyotime, Shanghai, China) was applied to determine the protein concentration of colon tissues for analysis of the concentration of MDA and SOD in the corresponding samples.

### 2.12. Cell Culture and Treatment

The RAW264.7 cell line was obtained from the State Key Laboratory for Diagnosis and Treatment of Infectious Diseases of the First Affiliated Hospital, Zhejiang University School of Medicine (Zhejiang, China). The cells were identified as mouse macrophages by flow cytometry (Figure S1). RAW264.7 cells were cultured with Dulbecco’s modified Eagle medium (DMEM) containing 1% streptomycin/penicillin and 10% fetal bovine serum (FBS) (Procell Life Science and Technology, Wuhan, China) in a humidified thermostatic incubator at 37 °C and 5% CO_2_. RAW264.7 cells were passaged every day. The colitis model in vitro was constructed using RAW264.7 cells treated with lipopolysaccharides (LPS) for 24 h. In our study, 5 × 10^6^ RAW264.7 cells were initially inoculated onto 6-well plates and treated with DMEM without FBS for 12 h before the treatment of LPS or (and) HT. In addition, the cells were pretreated with HT for two hours before the stimulation of LPS for another 24 h.

### 2.13. Cell Viability

Initially, 5 × 10^3^ RAW264.7 cells were seeded onto the 96-well plate, and the next day, the cells were incubated with different concentrations of HT (0, 12.5, 25, 50, 100, 200, 400, and 600 μM) for 24 h. Then, the media with HT was replaced by 110 μL of new media containing 10 μL of the Counting Kit-8 (CCK8) reagent (Beyotime, Shanghai, China), followed by incubation in a humidified thermostatic incubator at 37 °C and 5% CO_2_ for two hours. The optical density (OD) value was detected at a wavelength of 450 mm in an Epoch microplate reader (Biotek, Wienuski, VT, USA) to choose the appropriate concentration of HT for treating colitis in vitro. Every sample was tested in three duplicated wells and this experiment was repeated three times.

### 2.14. Intracellular Reactive Oxygen Species (ROS), Lipid Peroxidation (LPD), and Mitochondrial Membrane Potential (MMP)

For the detection of the level of intracellular ROS of RAW264.7 cells, a reactive oxygen species assay kit (Biosharp, Hefei, China) with a Diacetyldichlorofluorescein (DCFH-DA) probe was used. Briefly, after the aforementioned treatment, the cells of each group were washed gently with cold Phosphate-Buffered Saline (PBS) and then incubated with 1 mL DMEM with a DCFH-DA probe (1:3000 dilution) for thirty minutes at 37 °C. Subsequently, the stained cells were washed with cold PBS three times. The level of intracellular ROS was detected by an Olympus IX73 fluorescence microscope (Olympus, Tokyo, Japan) at the excitation wavelength of 460–490 nm and the emission wavelength of ≥510 nm.

To determine the level of intracellular LPD of RAW264.7 cells, BODIPY™ 581/591 C11 (Thermo Fisher Scientific, Waltham, MA, USA) was applied. The RAW264.7 cells were treated as aforementioned and collected, followed by the incubation of 0.5 mL of DMEM with a C11-BODIPYTM probe (1:2000 dilution) at a final concentration of 5 µmol/L for thirty minutes at 37 °C and then washed three times with cold PBS. The level of intracellular LPD was detected at FITC and PE channels by flow cytometry (CytoFLEX; Beckman Coulter, S. Kraemer Blvd, Brea, CA, USA).

To determine the injury of mitochondria, the MMP of RAW264.7 cells was detected with the enhanced mitochondrial membrane potential assay kit with JC-1 (Biosharp, Hefei, China) according to the instructions. Specifically, the JC-1 probe was diluted with JC-1 dyeing buffer (1:200). After incubation with the diluted JC-1 probe for 20 min at 37 °C, the cells were washed three times with cold PBS. MMP was detected at FITC and the PE channel by flow cytometry (CytoFLEX; Beckman Coulter, S. Kraemer Blvd, Brea, CA, USA) and the ratio of JC-1 aggregates (FITC-PE+ cells) against monomers (FITC+PE- cells) represented the MMP. In order to make the results of MMP more visible, the images of JC-1 probe were taken by an Olympus IX73 fluorescence microscope (Olympus, Japan). Green, fluorescent photos indicating the monomers were taken at an excitation wavelength of 460–490 nm and an emission wavelength of ≥510 nm; red fluorescent photos indicating the aggregates were taken at an excitation wavelength of 530–550 nm and an emission wavelength of ≥575 nm.

The experiments of ROS, LPD, and MMP were repeated three times.

### 2.15. Data Analysis

All statistical analyses in this study were processed on SPSS 20.0 software. For the assessment of statistical differences among the three groups, the Kruskal–Wallis test or One-way analysis of variance (ANOVA) was applied. The *p*-value of multiple comparisons was corrected using the LSD method. The data in images are shown as mean ± SEM or SD, and *p* < 0.05 is regarded as an indication of significance. GraphPadPrism 9.5 was used for plotting.

## 3. Results

### 3.1. Hesperetin Alleviated DSS-Induced Colitis in Mice

**Figure 1:**
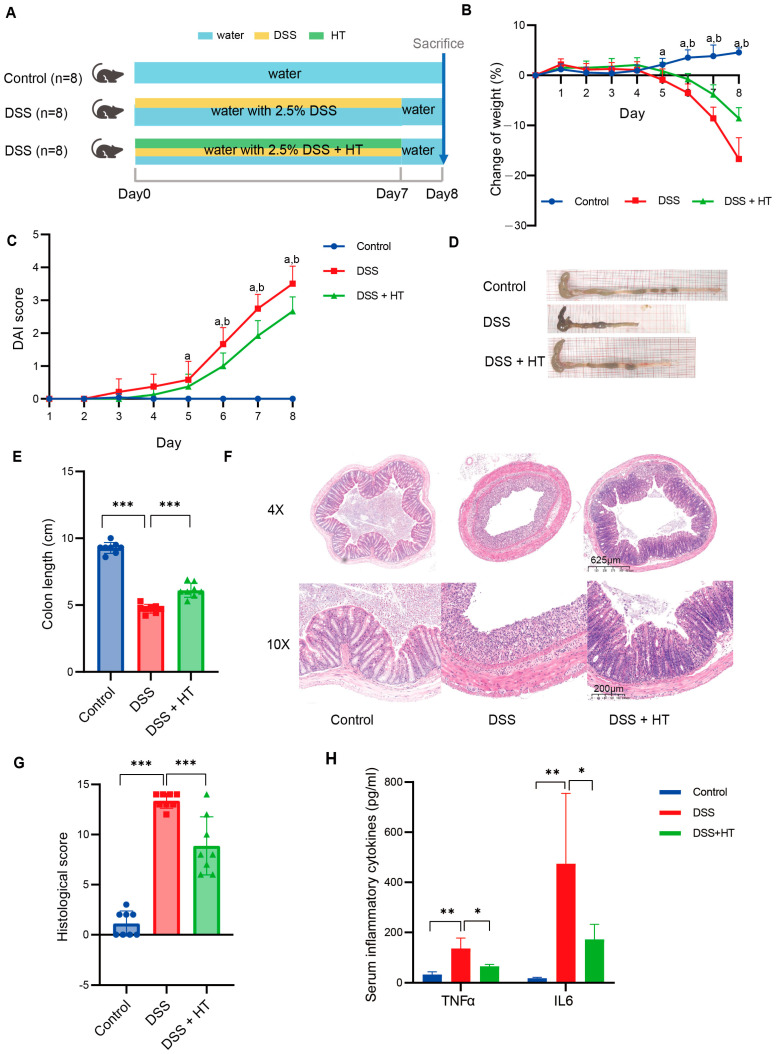
HT-improved indicators of colitis severity. (**A**) The design of animal experiments. (**B**) The change in body weight of mice during the experiment. (**C**) The DAI score of mice during the experiment. (**D**,**E**) The representative pictures of colon in each group (**D**) and the measurement of the colon length (**E**). (**F**,**G**) The representative pictures of the pathological change in each group shown by HE staining (**F**) and the histological score (**G**). (**H**) The level of TNFα and IL6 in serum of mice. DSS, dextran sodium sulfate; HT, hesperetin; DAI, disease activity index. The data in image (**B**,**C**,**E**,**G**) are shown as mean ± SD; the data in image H are shown as mean ± SEM. *, *p* < 0.05; **, *p* < 0.01; ***, *p* < 0.001; a, *p* < 0.05, DSS vs. control; b, *p* < 0.05, DSS + HT vs. DSS.

The detailed design of the animal experiment is shown in Figure 1A. No significant difference in weight was detected among mice of the three groups at the beginning of the experiment (Figure S2). With the drinking water supplemented with 2.5% DSS, the mice showed a decrease in bodyweight from the fifth day compared to the mice receiving normal water; meanwhile, the mice in the DSS + HT group showed a significantly smaller reduction in bodyweight in comparison to the DSS group from the sixth day (Figure 1B). The DAI score of mice in the DSS and DSS + HT groups increased steadily until the end of the experiment. With the supplement of HT, a significant decrease in DAI score was observed in the DSS + HT group in contrast with the DSS group from the sixth day (Figure 1C). Moreover, HT significantly improved the colon shortening (Figure 1D); the representative pictures of the colon are shown in Figure 1E. For histopathology, HE staining showed obvious colon lesions in the DSS-treated mice, which indicated the successful construction of the colitis model. In the DSS + HT group, less inflammatory infiltration and gland destruction with a smaller range of lesions were observed compared with the DSS group (Figure 1F–H). Mice in the DSS + HT group also manifested significantly lower levels of inflammatory cytokines (TNFα and IL6) in serum vs. the DSS-treated mice.

### 3.2. Combination of Bioinformatics, Pharmacology Network and Molecular Docking Revealed the Potential Targets of HT in UC

**Figure 2:**
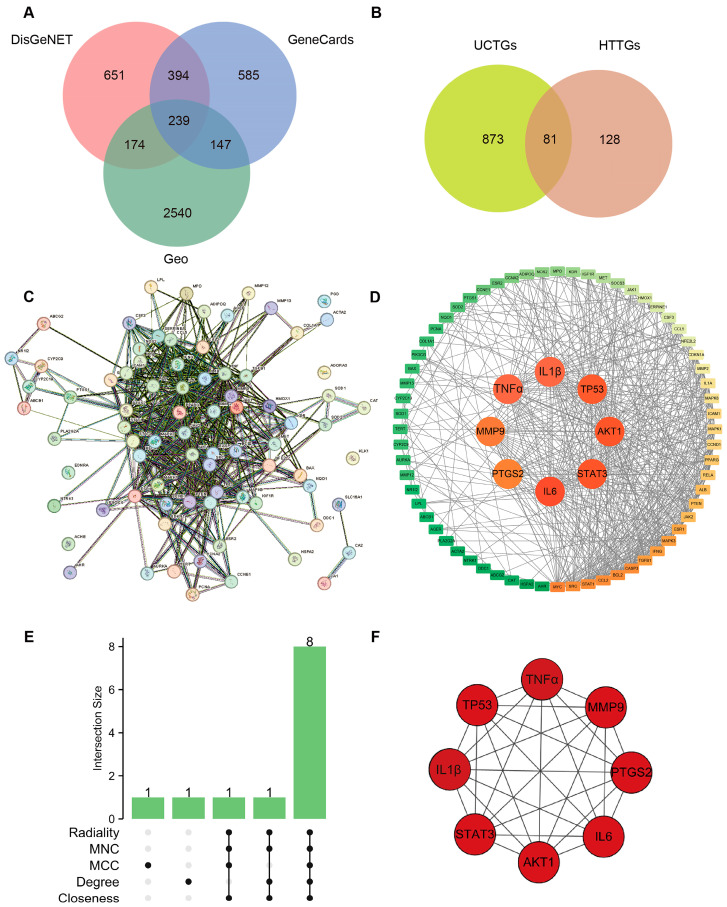
Verification of targets of HT in the treatment of UC. (**A**) Identification of target genes in the treatment of UC. The predicted genes existing in more than one of DisGeNET, GeneCards, and GEO databases were defined as UCTGs. (**B**) Identification of UCHTTGs. Target genes of HT were acquired from the ETCM, CTD, and STP, respectively, and HTTGs were identified with the duplicates removed. The intersection of HTTGs and UCTGs was identified as UCHTTGs. (**C**,**D**) The establishment of PPI network via the STRING database (**C**) and visualized by the Cytoscape software (**D**). (**E**) The core targets of HT in the treatment of UC shown by Upset diagram. (**F**) The interrelationship of hub genes of HT in the treatment of UC. UC, ulcerative colitis; HT, hesperetin; UCTGs, target genes in the treatment of UC; HTTGs, target genes of HT; UCHTTGs, target genes of HT in the treatment of UC; GEO, Gene Expression Omnibus; ETCM, Encyclopedia of Traditional Chinese Medicine; CTD, Comparative Toxicogenomics Database; STP, SwissTargetPrediction; PPI, Protein–Protein Intersection.

**Figure 3:**
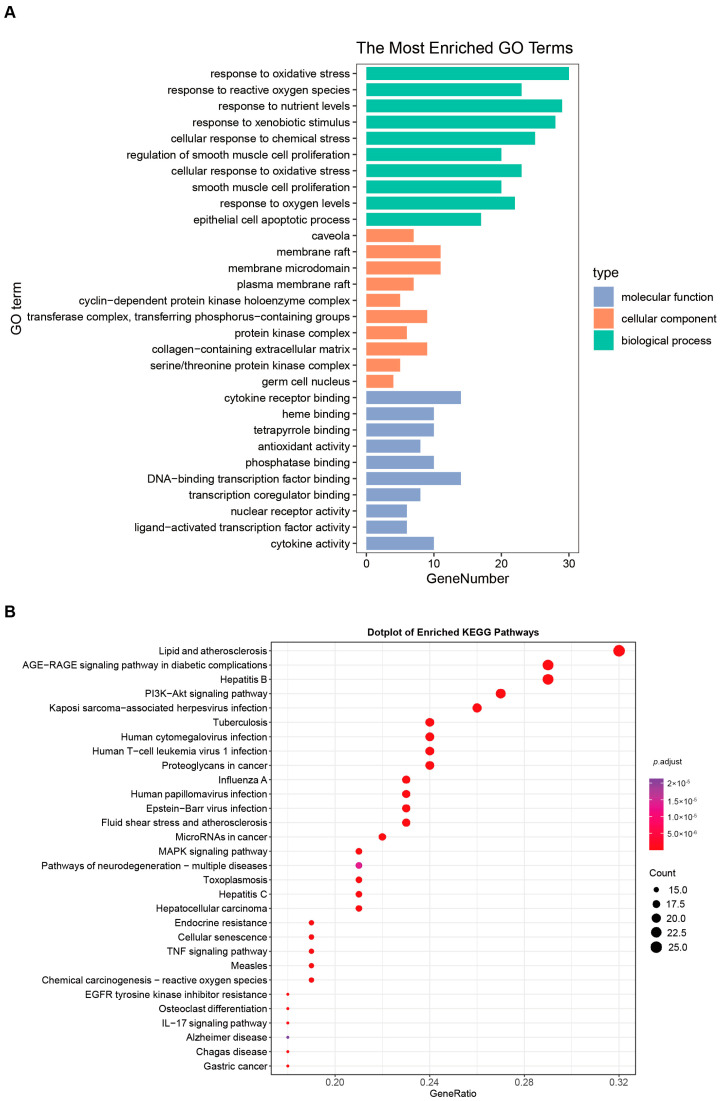
GO functional annotation and KEGG pathway enrichment. (**A**) The bar plot of the ten most significant biological processes influenced by HT in the treatment of UC based on the GO functional annotation. (**B**) The bubble plot of the top 30 enriched KEGG terms of pathways. The bubble size represents the number of genes, and the color of the bubble represents the *p*-value. GO, Gene Ontology; KEGG, Kyoto Encyclopedia of Genes and Genomes; UC, ulcerative colitis; HT, hesperetin.

**Figure 4:**
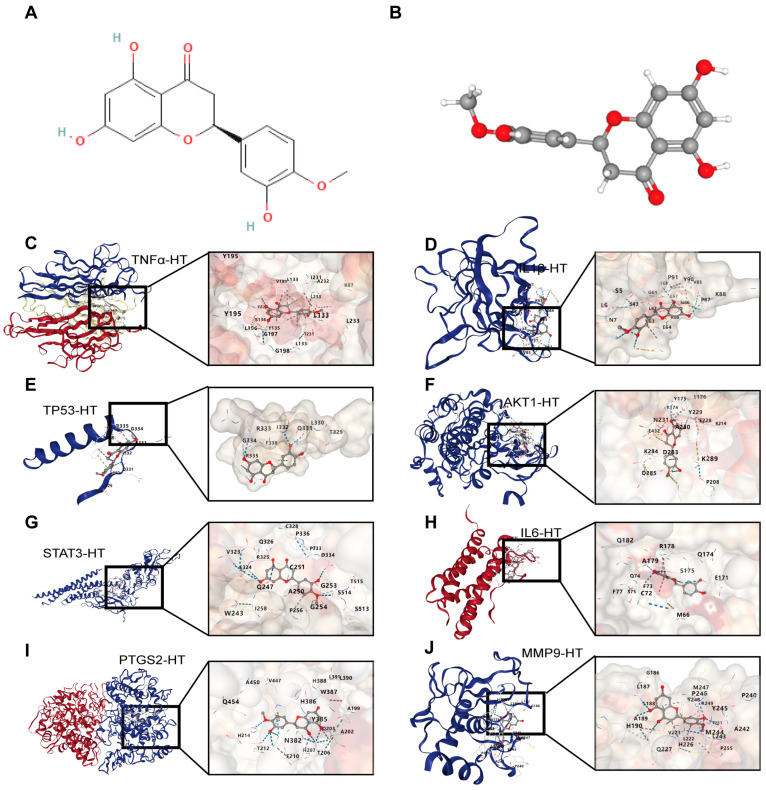
The visualization of molecular docking between HT and its target proteins in UC. (**A**,**B**) The structure of HT in 2D (**A**) and 3D (**B**) forms. (**C**) The interaction modes of HT and TNFα. (**D**) The interaction modes of HT and IL1β. (**E**) The interaction modes of HT and TP53. (**F**) The interaction modes of HT and AKT1. (**G**) The interaction modes of HT and STAT3. (**H**) The interaction modes of HT and IL6. (**I**) The interaction modes of HT and PTGS2. (**J**) The interaction modes of HT and MMP9. UC, ulcerative colitis; HT, hesperetin.

For UCTGs, bioinformatics was first applied to screen differential genes in GEO datasets GSE65114 and GSE87466, and then target genes of UC were downloaded from DisGeNET and GeneCards. In total, 954 genes that existed in more than one database were determined as UCTGs. A total of 209 HTTGs were identified from the ETCM, CTD, and STP databases, with the deletion of repeated ones. Eighty-one UCHTTGs were acquired by the overlap of UCTGs and HTTGs (Figure 2A,B). The PPI network of the corresponding UCHTTG protein was established by STRING 12.0 [44], and then visualized with Cytoscape 3.9.1 [45] (Figure 2C,D). Using the plug-in cytoHubba [45], eight hub genes (*TNFα*, *IL1β*, *TP53*, *AKT1*, *STAT3*, *IL6*, *PTGS2*, and *MMP9*) were identified as the key targets of HT in UC (Figure 2E,F). The GO analysis showed that HT mainly influenced the response to oxidative stress of the biological process (Figure 3A). Meanwhile, KEGG enrichment revealed that HT was involved in various pathways, such as PI3K-Akt, MAPK, and TNF signaling pathways (Figure 3B). To verify the key targets of HT in UC, molecular docking was used to predict the binding cavity volume, center of cavities, docking size, and the Vina Score (kcal/mol) between HT and the target protein. Theoretically, binding energy of less than −5.0 kcal/mol indicated by the Vina score suggests good binding activity between the ligand and protein [50]; the lower the Vina score is, the more likely hesperetin would bind to the protein. It seems that HT could bind to the eight core targets (TNFα, IL1β, TP53, AKT1, STAT3, IL6, PTGS2, and MMP9) with a Vina score pf < −5.0 kcal/mol (Table 3). The docking was visualized on the CB-DOCK2 website (Figure 4A–J).

### 3.3. Verification of Target Genes of HT In Vivo

**Figure 5:**
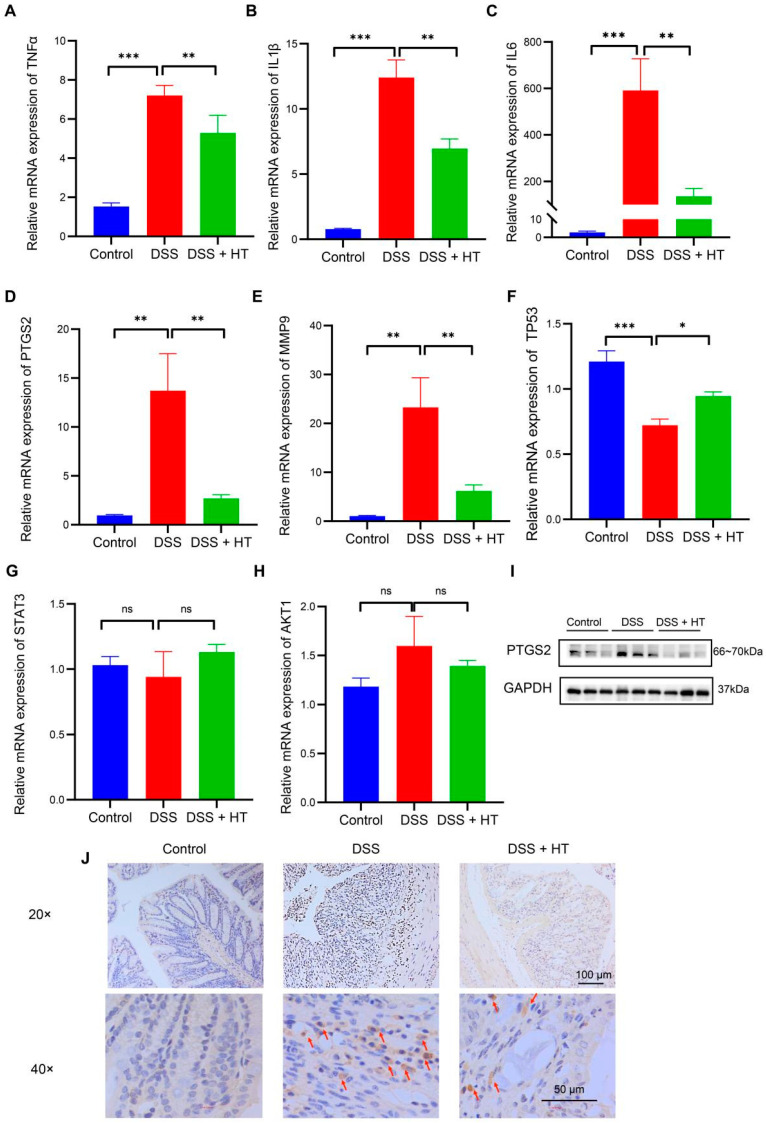
The verification of the core targets of HT in DSS-induced colitis. (**A**–**H**) The influence of HT on the mRNA expression of TNFα (**A**), *IL1β* (**B**), *IL6* (**C**), *PTGS2* (**D**), *MMP9* (**E**), *TP53* (**F**), *STAT3* (**G**), and *AKT1* (**H**). (**I**,**J**) The protein expression of PTGS2 protein detected by WB (**I**) and IHC (**J**). The arrows represent the PTGS2-positive cells. HT, hesperetin; WB, Western blot; IHC, immunohistochemistry. The data in images are shown as mean ± SEM. *, *p* < 0.05; **, *p* < 0.01; ***, *p* < 0.001; ns, no significant difference.

An acknowledged animal model of UC was used to verify the predictive targets of HT in colitis. At the level of mRNA, the relative expression of *TNFα*, *IL1β*, *IL6*, *MMP9*, and *PTGS2* increased significantly in DSS-induced colitis compared with the control group. In contrast, HT markedly downregulated the expression of the five genes in DSS-induced colitis (Figure 5A–E). Meanwhile, *TP53* was decreased in DSS-induced colitis in contrast with the normal mice and HT restored the mRNA expression of *TP53* (Figure 5F). Additionally, no significant change in *AKT1* or *STAT3* was observed among the three groups (Figure 5G,H).

### 3.4. HT Suppressed Ferroptosis in Colitis In Vivo

**Figure 6:**
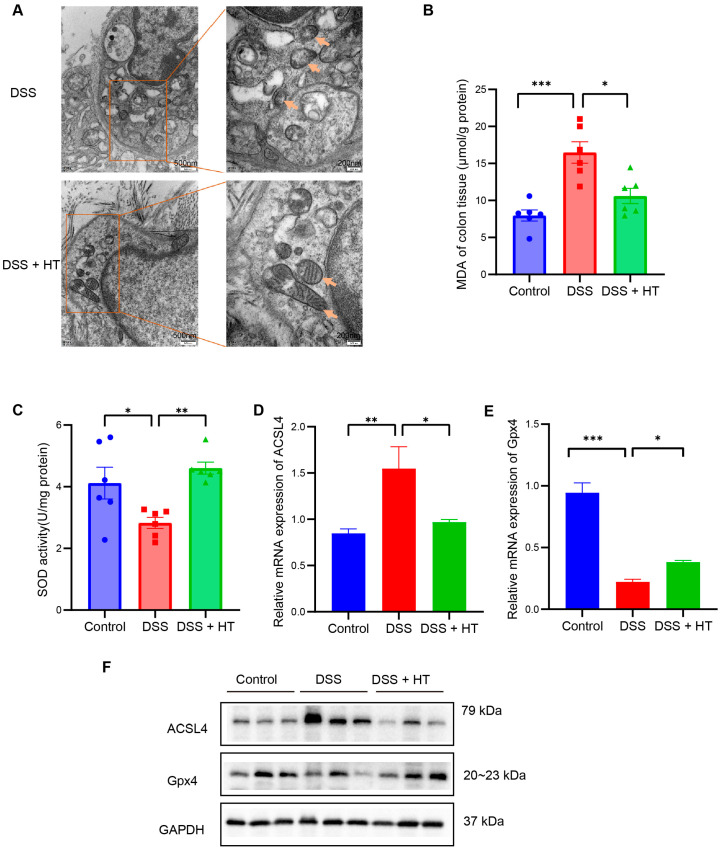
HT suppressed ferroptosis in DSS-induced colitis. (**A**) The TME pictures of colon show rupture of mitochondrial outer membrane, with disappearance of mitochondrial cristae in DSS-induced colitis. The supplementation of HT improved the mitochondrial morphology with more clear mitochondrial cristae and outer membrane. Arrows indicate the representative change of mitochondria. (**B**) HT reduced MDA in colitis. (**C**) HT restored the activity of SOD in colitis. (**D**–**F**) HT decreased the expression of ACSL4 and increased Gpx4 at the mRNA level (**D**,**E**) and protein level (**F**), respectively. TME, Transmission Electron Microscope; MDA, Malondialdehyde; SOD, Superoxide Dismutase; DSS, dextran sodium sulfate; HT, hesperetin. The data in images are shown as mean ± SEM. *, *p* < 0.05; **, *p* < 0.01; ***, *p* < 0.001.

PTGS2 is a well-known marker of the occurrence of ferroptosis, and ferroptosis is closely related to oxidative stress [51]. We found that HT significantly lowered the colonic mRNA expression of PTGS2 in DSS-induced colitis and influenced the response to oxidative stress of the biological process. Therefore, we assumed that HT might modulate ferroptosis in colitis. We first determined the influence of HT on the protein level of PTGS2 in colitis. The Western blot and immunohistochemistry examination showed that HT reduced the expression of PTGS2 in the colon with colitis, which indicated that HT suppressed ferroptosis in colitis (Figure 5I,J). Further, other markers of ferroptosis were detected. The representative TME pictures of the colon manifested the rupture of the mitochondrial outer membrane, with the disappearance of mitochondrial cristae in DSS-induced colitis. The treatment of HT improved the mitochondrial damage with more clear mitochondrial cristae and continuous outer membrane (Figure 6A). In addition, compared with the control, MDA was increased while SOD was decreased in the colon with colitis. HT reversed the level of MDA and SOD in DSS-induced colitis (Figure 6B,C), demonstrating that HT inhibited lipid peroxidation and ROS. Moreover, the supplementation of HT significantly downregulated the increased ACSL4 in DSS-induced colitis while it upregulated the decreased Gpx4 both in mRNA and protein expression (Figure 6D–F), suggesting that HT suppressed ferroptosis in colitis in vivo.

### 3.5. HT Suppressed Ferroptosis in LPS-Induced RAW264.7 Cells

**Figure 7:**
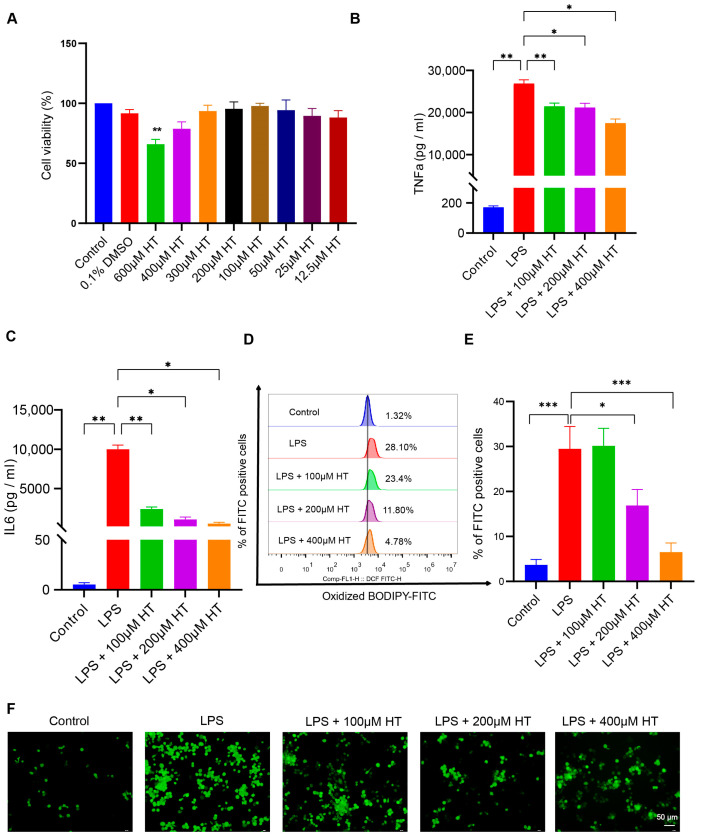

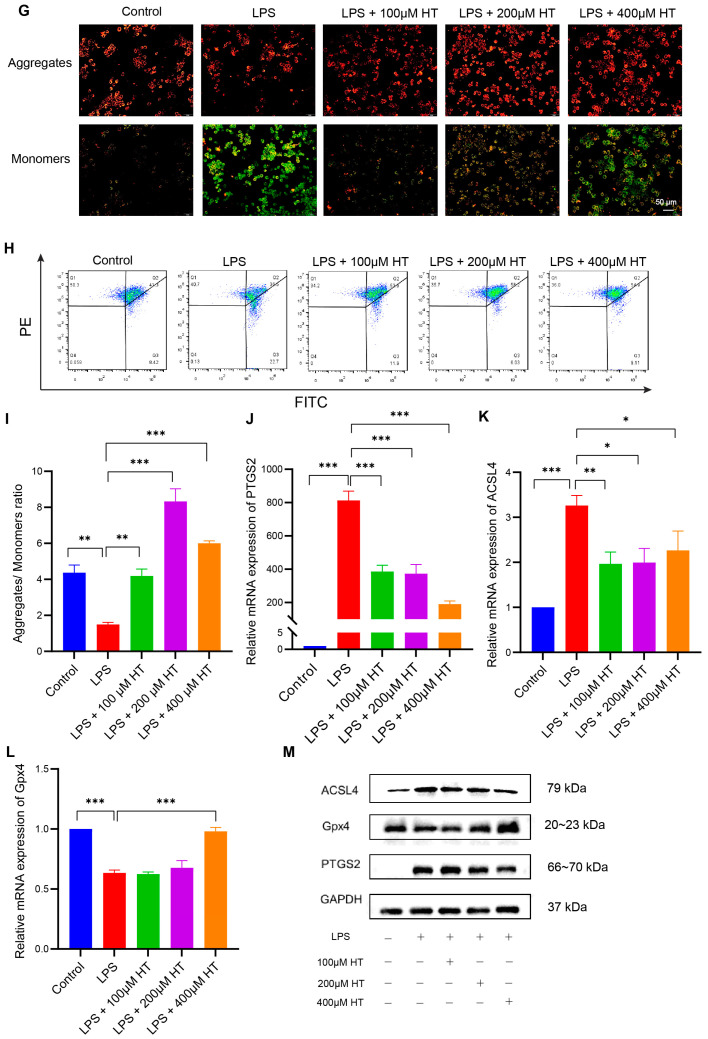
HT inhibited ferroptosis in LPS-induced RAW264.7 cells. (**A**) The choice of the appropriate concentrations of HT for the treatment of RAW264.7 cells by CCK-8 examinations. (**B**,**C**) HT significantly decreased the level of TNFα (**B**) and IL6 (**C**) in the supernatant of cells incubated with LPS. (**D**,**E**) The detection of the lipid peroxidation in LPS-treated RAW RAW264.7 cells with the application of the BODIPY probe examined by flow cytometer (**D**) and the percentage of FITC-positive cells (**E**). (**F**) The representative pictures of cellular ROS with the DCFH-DA probe. (**G**) The influence of HT on the MMP with the JC-1 probe examined by a fluorescence microscope. The red fluorescence (PE) represents aggregates of the JC-1 probe and the green fluorescence (FITC) represents monomers. (**H**,**I**) The influence of HT on the MMP with the JC-1 probe examined by flow cytometer. The cells in Q2 represent aggregates of the JC-1 probe and the cells in Q4 represent monomers (**H**). The MMP was indicated by the ratio of aggregates to monomers (**I**). (**J**–**M**) The supplementation of HT regulated the expression of PTGS2, ACSL4, and Gpx4 at the mRNA (**J**–**L**) and protein levels (**M**). LPS, lipopolysaccharides; FITC, Fluorescein Isothiocyanate; DCFH-DA, Dichlorodihydrofluorescein diacetate; MMP, mitochondrial membrane potential; HT, hesperetin. The data in images are shown as mean ± SEM. *, *p* < 0.05; **, *p* < 0.01; ***, *p* < 0.001.

To establish the cellular inflammation model, LPS was used to stimulate the RAW264.7 cells for 24 h. Incubation with LPS led to an intracellular overload of iron in the RAW264.7 cells [52]. Different concentrations (0.005, 0.01, 0.05, 0.1, 0.2, 0.3, 0.5, 1, 2, and 5 μg/mL) of LPS were applied to screen an appropriate concentration to induce the colitis model in vitro. Compared with the normal control, *TNFα*, *IL6*, and *ACSL4* mRNA expression increased with ≥0.005 μg/mL LPS; *IL1β* and *PTGS2* mRNA expression increased with ≥0.01 μg/mL LPS; IL10 mRNA expression increased with ≥2 μg/mL LPS (Figure S4A–F). Finally, a concentration of 0.2 μg/mL LPS was adapted for the following experiments. The CCK8 experiment was performed to select a suitable HT concentration. No significant difference in cell viability was found between HT-treated cells and the control cells at the HT concentration ≤ 400 μM (Figure 7A).

After coculture with HT and LPS for 24 h, HT (100, 200, 400 μM) significantly decreased TNFα and IL6 in the supernatant of LPS-treated cells (Figure 7B,C), which indicated that HT inhibited the inflammation of colitis in vitro. Meanwhile, a marked decrease in lipid peroxidation and ROS, together with an obvious increase in MMP, was observed in HT-LPS-treated RAW264.7 cells in comparison with LPS-stimulated RAW264.7 cells (Figure 7D–I). Furthermore, HT regulated ferroptosis-related genes and proteins. In detail, HT downregulated ACSL4 and PTGS2 mRNA and protein, while it upregulated Gpx4 mRNA and protein (Figure 7J–M), suggesting that HT suppressed ferroptosis in colitis in vitro.

### 3.6. HT Regulated the Gut Microbiota in DSS-Induced Colitis

**Figure 8:**
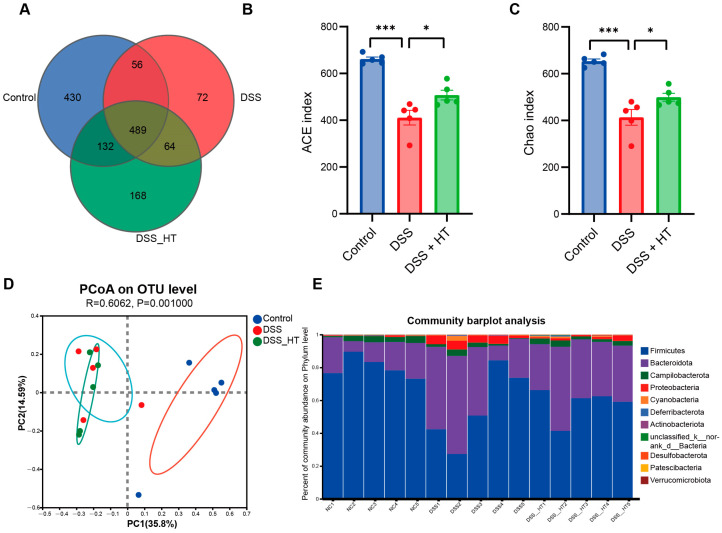

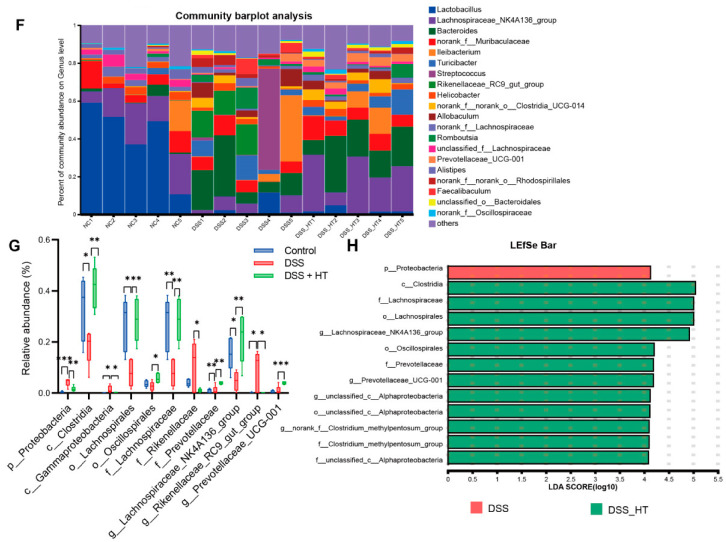
HT modulated gut microbiota in colitis (**A**) OTUs of gut microbiota in each group. (**B**,**C**) HT influenced α diversity of gut microbiota in mice with colitis. Ace index (**B**) and chao index (**C**) at the OUT level. (**D**) The effect of HT on β diversity of gut microbiota in mice with colitis with Anosim at the level of OUT. (**E**,**F**) The prominent phyla (**E**) and genera (**F**) in the three groups. (**G**) The bar plot of the relative abundance of bacteria influenced by HT. (**H**) Lefse reveals the distinct gut bacteria between DSS and DSS + HT groups, with LDA ≥ 4. DSS, dextran sodium sulfate; HT, hesperetin; Anosim, analysis of similarities; LDA, linear discriminant analysis. The data in images are shown as mean ± SEM. *, *p* < 0.05; **, *p* < 0.01; ***, *p* < 0.001.

To determine how HT affected the gut microecology in DSS-induced colitis, 16S rRNA gene sequencing was applied to unveil the change in gut microbiota. The gut microbiota of the mice treated with DSS were significantly different from that of the control group. Specifically, the DSS group manifested fewer operational taxonomic units (OTUs) and a marked decrease in α diversity compared with the control group. In contrast, the treatment of HT reversed this decline slightly with a higher value of the ACE index (*p* < 0.001) and the Chao index (*p* < 0.05) (Figure 8A–C). A marked difference in β diversity was also observed among the three groups (Figure 8D). Figure 8E,F shows dissimilar compositions at the phylum and genus levels of gut microbiome among the three groups, respectively, indicating that oral DSS obviously affected the gut microbiota while the supplementation of HT regulated the composition of microbiota in the colon. To be exact, the abundance of Proteobacteria and Gammaproteobacteria decreased in the DSS + HT group compared with the DSS group. Several bacteria closely associated with SCFA production, namely Lachnospirales, Oscillospirales, Lachnospiraceae, Prevotellaceae, Prevotellaceae_UCG-001, and Lachnospiraceae_NK4A136_group, were restored by HT in DSS-induced colitis. In addition, the relative abundance of Clostridia, Rikenellaceae, and Rikenellaceae_RC9_gut_group was returned to almost the same levels as those in the normal mice (Figure 8G,H).

## 4. Discussion

HT has been demonstrated to alleviate UC by downregulating oxidative stress, inflammation, and apoptosis and improving the gut barrier in experimental colitis [53,54]. However, limited studies centered on the protective effect and relative mechanisms of HT in UC have been conducted. Here, we found that supplementation with HT alleviated the DAI, pathohistological severity, and inflammation in DSS-induced colitis, with the inhibition of ferroptosis and modulation of gut microbiota.

Consistent with previous studies [54], we demonstrated that HT improved weight loss, hematochezia, diarrhea, colon shortening, and inflammation, suggesting the therapeutic potential of HT in UC. To unveil the molecular targets of HT in UC, experiments combined with informatics, pharmacology network, and molecular docking were used. Interestingly, PTGS2, a marker of ferroptosis [51], was identified as a hub gene of HT in UC. Ferroptosis was recently recognized as a therapeutic target for UC and other intestinal diseases related to inflammation [55], which supported our findings. PTGS2 increased significantly in the DSS-induced colitis and decreased via the supplementation of HT. Using the GO enrichment analysis, HT was demonstrated to influence the response to oxidative stress of biological processes in UC. Since ferroptosis is a programmed death closely related to oxidative stress and lipid peroxidation [24], we assumed that HT might suppress ferroptosis in UC. Compatibly, HT regulated ferroptosis-related genes and proteins in DSS-induced colitis. ACSL4 was downregulated and Gpx4 was upregulated by HT treatment in colitis. Improved mitochondrial morphology of the colon tissue detected by TEM was observed in the DSS + HT group compared with the DSS group, with clearer mitochondrial crista and less of an abnormal mitochondrial structure. Additionally, HT supplementation reduced lipid peroxidation and enhanced the antioxidation system in colitis, as shown by colonic MDA and SOD, respectively. This evidence demonstrated that HT inhibited ferroptosis in DSS-induced colitis. It is unclear whether HT is a ferroptosis inhibitor so far. However, HT was found to promote wound healing by reducing ferroptosis in a rat diabetic model [56], and other flavonoids such as hesperidin, naringenin, and quercetin were shown to modulate ferroptosis in various diseases [57]. The findings of other researchers agree with our hypothesis to some extent. To further verify our observation, an in vitro colitis model was applied, and the treatment of HT inhibited inflammation and ferroptosis in LPS-treated RAW264.7 cells by increasing MMP and reducing cellular lipid peroxidation and ROS. In line with our study, several studies proved that the reduction in macrophage ferroptosis was helpful for the improvement of experimental colitis [58,59].

Our current study demonstrated that HT modulated the composition of the gut microbiome in DSS-induced colitis. The gut microbiome significantly influences the homeostasis of the host and has been proven to be an emerging target for a variety of diseases, such as gastroenterology diseases, neurological disorders, and cardiovascular diseases [60]. The gut microbiota of UC patients was different from the healthy pat, highlighted by the obvious decreased diversity of gut microbiota [61], which was in line with our findings in DSS-induced rodent colitis. Intestinal flora was recognized to play a key role in the pathogenesis and progression of UC [62]. It was shown that HT reshaped the gut microbiome and upregulated the level of short-chain fatty acids in healthy rats [63]. Additionally, HT was found to be a potential supplement for preventing mastitis by modulating the microbiota and enhancing the blood–milk barrier [64], indicating that HT has a prebiotic-like effect in vivo. However, whether HT regulated the gut flora in colitis is unknown. Here, HT increased the diversity of gut microbiota in the experimental colitis with the elevation of the ACE and Chao indices, indicating that HT has prebiotic and therapeutic potential for colitis [65]. In our study, HT decreased the relative abundance of pro-inflammatory bacteria (Proteobacteria and Gammaproteobacteria) in colitis while restoring the relative abundance of SCFA-producing bacteria (Lachnospirales, Lachnospiraceae, Lachnospiraceae_NK4A136_group, Oscillospirales, Prevotellaceae, and Prevotellaceae_UCG-001), which were negatively associated with diseases [66,67,68,69,70]. Although HT decreased the relative abundance of f_Rikenellaceae and g_Rikenellaceae_RC9_gut_group, accompanied by increased c_Clostridia, which is different from the findings from previous studies [71,72], it was reasonable that HT supplementation rendered the relative abundance of these bacteria in colitis similar to the normal control.

## 5. Conclusions

In conclusion, to decipher the mechanisms behind how HT protects against UC, we performed microbiome, informatics, pharmacology network, and molecular docking, together with experimental validation. It was shown that HT significantly ameliorated the colonic pathological change, symptoms of colitis, and inflammation responses through various targets and pathways. Interestingly, we confirmed that HT alleviated experimental colitis by suppressing ferroptosis in vivo and in vitro. Furthermore, HT regulated the composition of gut microbiota in DSS-induced acute colitis, which suggested HT as a potential prebiotic. Therefore, HT might be a beneficial nutritional supplement for the treatment of UC.

## Figures and Tables

**Table 1:** The list of websites of databases used in this study.

Database	Website
ETCM	http://www.tcmip.cn/ETCM/
CTD	https://ctdbase.org/
STP	http://swisstargetprediction.ch/
Pubchem	https://pubchem.ncbi.nlm.nih.gov/
DisGeNET	https://www.disgenet.org/
GeneCards	https://www.genecards.org/
GEO	https://www.ncbi.nlm.nih.gov/geo/
CB-DOCK2	https://cadd.labshare.cn/cb-dock2/php/index.php
jvenn	https://jvenn.toulouse.inrae.fr/app/example.html
STRING	https://cn.string-db.org/
PDB	https://www.rcsb.org/
Xiantao Academic Tools	https://www.xiantaozi.com/products

**Table 2:** The primers used in the RT-qPCR.

Gene Symbol	Forward(F)/Reverse(R)	Sequence
TNFα	F	CAGGCGGTGCCTATGTCTC
	R	CGATCACCCCGAAGTTCAGTAG
IL6	F	GAGGATACCACTCCCAACAGACC
	R	AAGTGCATCATCGTTGTTCATACA
IL1β	F	GCCACCTTTTGACAGTGATGAG
	R	ATGTGCTGCTGCGAGATTTG
STAT3	F	AGAACCTCCAGGACGACTTTG
	R	TCACAATGCTTCTCCGCATCT
PTGS2	F	TTCCAATCCATGTCAAAACCGT
	R	AGTCCGGGTACAGTCACACTT
MMP9	F	GGACCCGAAGCGGACATTG
	R	CGTCGTCGAAATGGGCATCT
AKT1	F	ATGAACGACGTAGCCATTGTG
	R	TTGTAGCCAATAAAGGTGCCAT
Gpx4	F	TGTGCATCCCGCGATGATT
	R	CCCTGTACTTATCCAGGCAGA
TP53	F	CCCCTGTCATCTTTTGTCCCT
	R	AGCTGGCAGAATAGCTTATTGAG
	R	GGACCAAAGACCTCCAGAATG
GAPDH	F	AGGTCGGTGTGAACGGATTTG
	R	TGTAGACCATGTAGTTGAGGTCA

**Table 3:** Docking results of HT and key target proteins in UC.

PDB ID	Protein Name	Vina Score(kcal/mol)	Cavity Volume	Center			Docking Size		
				x	y	z	x	y	z
7JRA	TNFα	−8.5	1364	−16	−3	−25	21	21	21
5R7W	IL1β	−7.5	189	40	9	57	22	22	22
1AIE	TP53	−6	13	8	17	9	22	22	22
7NH5	AKT1	−7.7	246	13	−2	4	22	22	22
6NJS	STAT3	−7.3	892	5	32	20	22	22	22
7NXZ	IL6	−6.3	116	−4	−12	2	22	22	22
5F19	PTGS2	−8.6	1234	32	37	18	22	22	32
6ESM	MMP9	−10.3	350	1	48	23	22	22	22

## Data Availability

The data supporting this study will be available from the corresponding author upon reasonable request.

## Supplementary Materials

The following supporting information can be downloaded at: https://www.mdpi.com/article/10.3390/nu16142343/s1. Figure S1: The validation of the RAW264.7 cell line, Figure S2: The weight of mice in the three groups at the beginning of the experiment, Figure S3: The identified target genes of UC in the GEO database by informatics, Figure S4: The screening of the concentration of LPS for the RAW264.7 cells, Table S1: Disease activity index (DAI) score, Table S2: Histopathological Score.
