# Supplementary material for: Hesperetin Alleviated Experimental Colitis via Regulating Ferroptosis and Gut Microbiota
Source: Nutrients. 2024 Jul 19;16(14):2343. doi: 10.3390/nu16142343 (PMC11279615; doi:10.3390/nu16142343)
Supplement: Supplementary file 1 [file nutrients-16-02343-s001.zip › nutrients-3064610-supplementary.pdf]

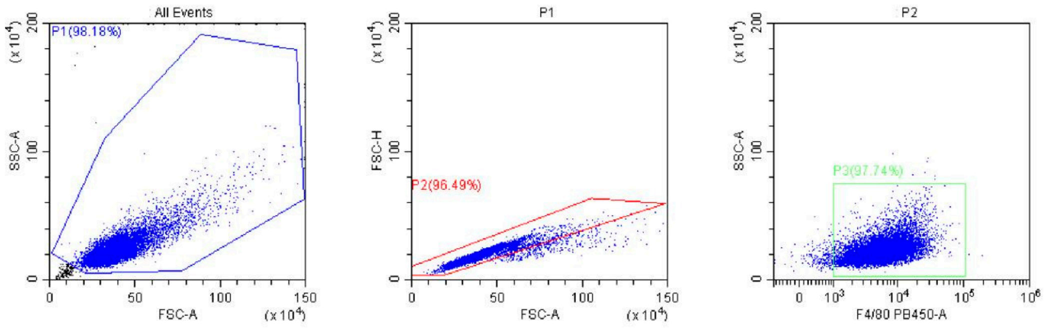

Figure S1. The validation of the RAW264.7 cell line.

The cells were stained with BV421 Rat Anti-Mouse F4/80 and the expression of F4/80 was detected by flow cytometry. The F4/80-positive cells were over 95%.

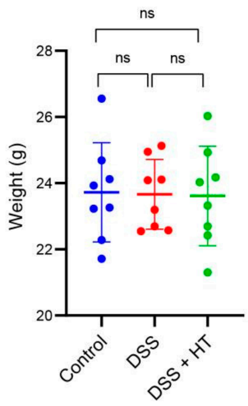

Figure S2. The weight of mice in the three groups at the beginning of the experiment

No significant difference was detected among the weight of mice in the three groups at the start of the experiment.

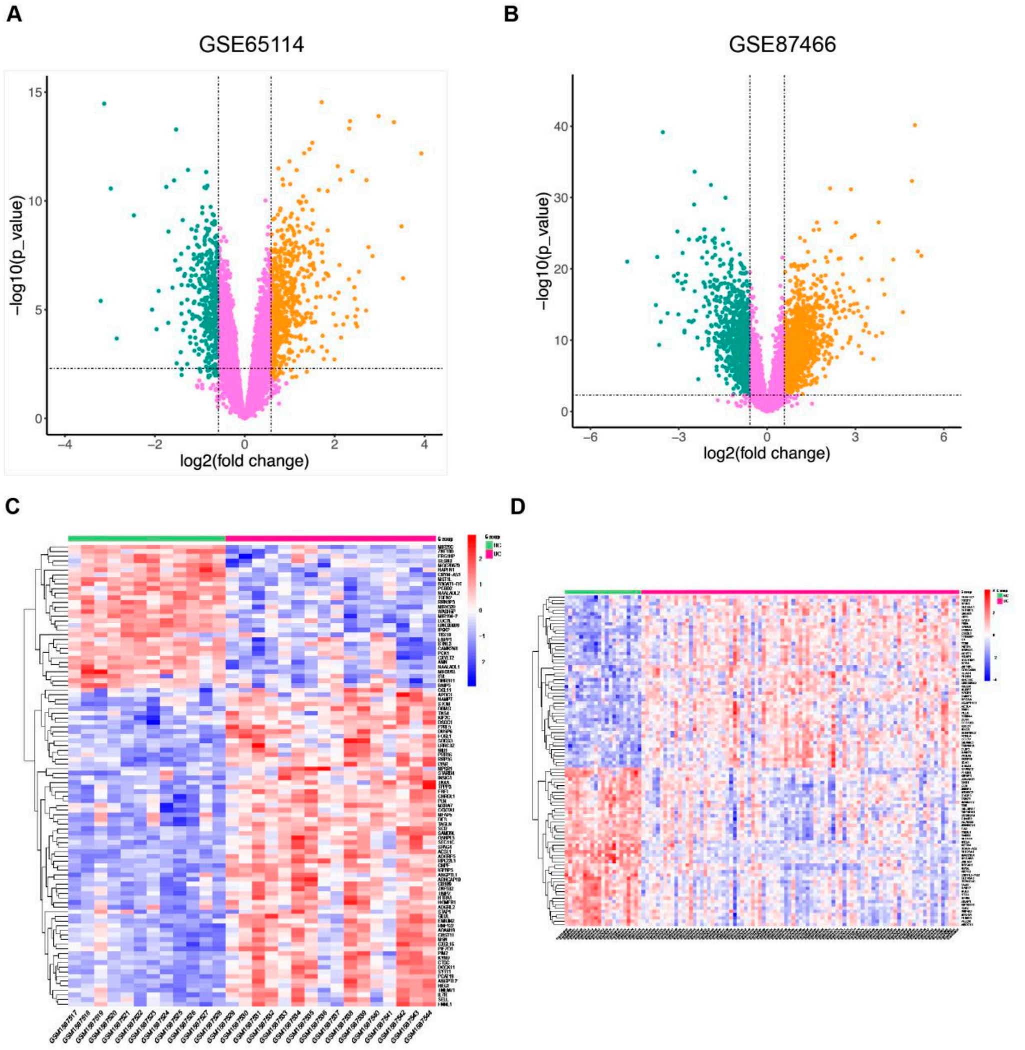

Figure S3. The identified target genes of UC in the GEO database by informatics

The dot plots (A-B) and hot plots (C-D) showed the differential expression genes with the criteria |fold change| ≥ 1.5 and adjusted.$p < 0.05$ of two datasets (GSE65114 and GSE87466) in the GEO database.

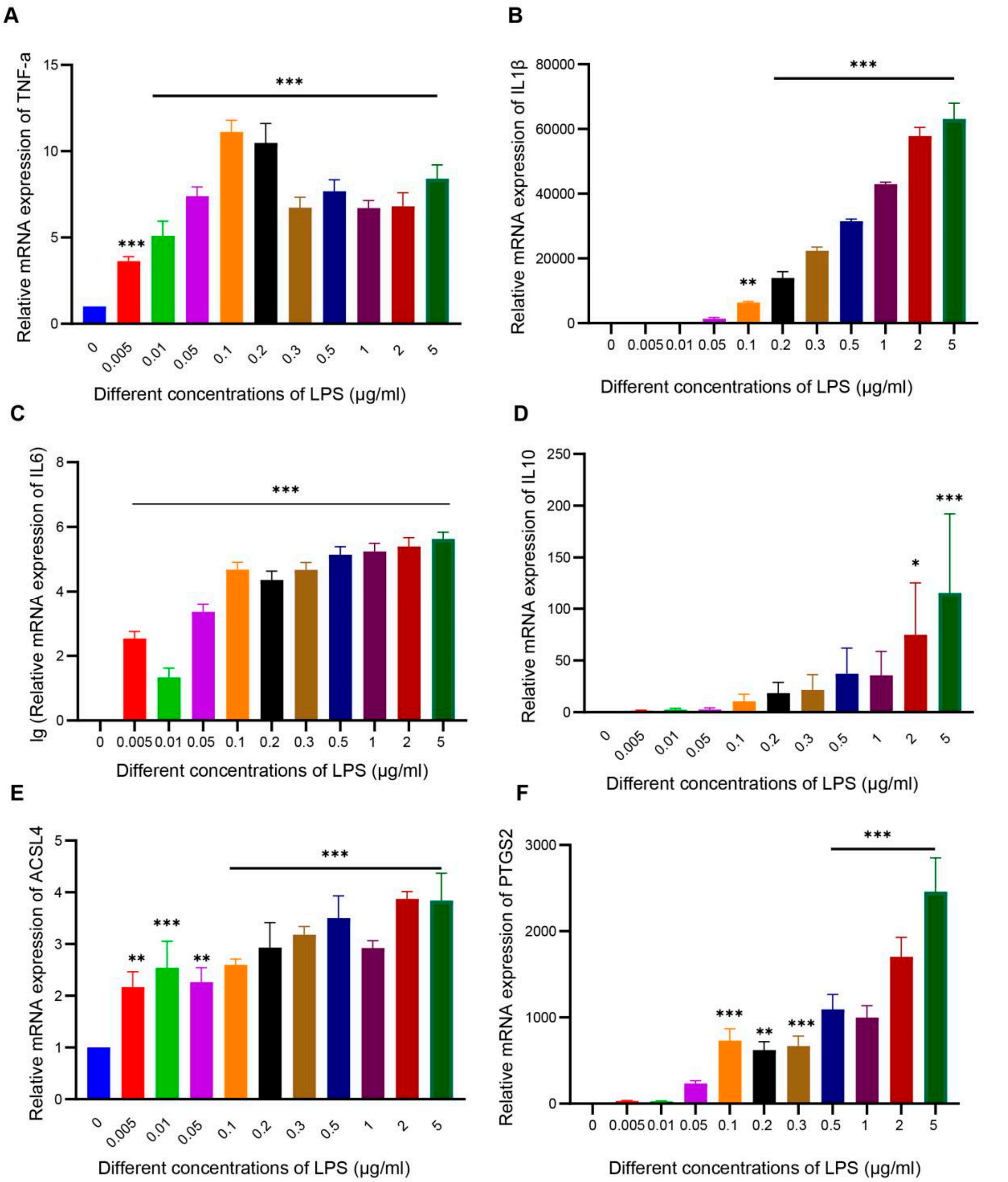

Figure S4. The screening of the concentration of LPS for the RAW264.7 cells

The bar plots showed that different concentrations of LPS affected the inflammatory response (TNFα, IL1β, IL6, and IL10) and the ferroptosis-related indicators (ACSL4 and PTGS2) in the RAW264.7 cells. LPS, lipopolysaccharides. *, $p < 0.05$; **, $p < 0.01$; ***, $p < 0.001$, compared with 0 μg/ml group.

Table S1. Disease activity index (DAI) score

Score	Body weight loss	Stool consistency	Fecal occult blood
0	Normal	Normal	Normal
1	1%-5%	-	-
2	5%-10%	Relaxed	Positive fecal occult blood
3	10%-20%	-	-
4	> 20%	Diarrhea	Gross blood stool

Table S2. Histopathological Score

Score	Inflammation	Depth of mucosal damage	Range of crypt damage	Range of pathological change
0	None	None	None	None
1	Mild	Mucous layer	1/3	1%-25%
2	Moderate	Submucosa	2/3	26%-50%
3	Severe	Muscularis and serosa	100%	51%-75%
4	-	-	Loss of the entire crypt and epithelial layer	76%-100%
